# Prioritizing knowledge translation in low- and middle-income countries to support pandemic response and preparedness

**DOI:** 10.1186/s12961-020-00670-1

**Published:** 2021-01-18

**Authors:** Yodi Mahendradhata, Anna Kalbarczyk

**Affiliations:** 1 Faculty of Medicine, Public Health and Nursing, Universitas Gadjah Mada, Yogyakarta, Indonesia; 2 Department of International Health, Johns Hopkins Bloomberg School of Public Health, 615 N Wolfe St., Baltimore, MD United States of America

**Keywords:** Knowledge translation, Evidence-based response, Pandemic preparedness, Policy-making

## Abstract

The COVID-19 pandemic has created urgent demand around the world for knowledge generation about a novel coronavirus, its transmission, and control, putting academic institutions at the frontline of politics. While many academic institutions are well poised to conduct research, there are well-documented barriers for these institutions, particularly in low- and middle-income countries (LMICs), to further conduct strategic synthesis and dissemination to promote knowledge utilization among policy-makers. These systemic barriers to knowledge translation (KT) pose significant challenges for academic institutions seeking to take advantage of unprecedented policy windows to inform evidence-based decision-making. Global health funding organizations should prioritize the support of academic institutions’ activities along the KT pathway, including both knowledge generation and strategic dissemination, to improve knowledge uptake for decision-making to improve health. Institutional capacity-building initiatives for KT have the potential to profoundly impact responses to this and future pandemics.

The COVID-19 pandemic has put academic institutions across the world on the frontlines of politics [1]. Difficult decisions are being made every day to manage imminent threats and determine how to keep the public informed about what is happening. Thus, how academic institutions generate and transfer knowledge to the real world is absolutely critical for countries in navigating the pandemic [2].

Academic institutions in low- and middle-income countries (LMICs) in particular face unique challenges in conducting knowledge translation (KT) activities to support the COVID-19 response in their contexts. The barriers and facilitators of conducting KT activities in low-resource settings have been documented in a few studies, and this area of research is growing [3, 4]. However, little has been discussed on KT activities conducted by academic institutions in LMICs during a complex global health emergency, such as the COVID-19 pandemic, and how these institutions can be more prepared in the future to conduct KT effectively in emergency and non-emergency situations.

## Barriers to conducting KT

Conducting KT activities, even in a non-emergency setting, already poses challenges to academic institutions in LMICs [3, 4]. These resource-constrained institutions experience (in more severe magnitude) the many well-documented barriers to conducting KT activities that have previously been described for high-income countries, including a lack of knowledge about what KT is and how to do it, limited resources and institutional support for KT, and the need for buy-in from members of leadership [5]. Three additional barriers which are more prominent in low-resource settings have recently been described: the complexity of the KT and policy processes and the need for soft skills; the role of institutional missions and incentives; and the value and challenges in developing robust internal and external networks. The complexity of the policy-making process requires soft skills to navigate and engage with policy-makers, which academics often lack and rarely receive training for. Concurrently, institutions lack the strategic support needed to continuously conduct KT, even if KT is integral to their institutional mission. The beneficial role of networks, both internal and external to the institution, facilitate acceptability of knowledge generated through research and its utilization in policy-making. These external networks are highly valuable for individuals and institutions but can be challenging to acquire.

In a recent commentary, Stewart et al., on behalf of the LMIC members of the COVID-19 Evidence Network to support Decision-making (COVID-END), highlighted the barriers that LMIC evidence communities have faced during COVID including a burgeoning demand that outweighs supply, limited access to technological hardware and software, and mistrust [6]. Common approaches to evidence synthesis, such as the systematic review, also take substantial time to complete (when done well), and in a pandemic setting, results can become quickly outdated [7]. The urgent demand for evidence can also create duplication of efforts and variable quality, particularly when conducted by those less familiar with KT methodologies [7].

The challenges of conducting KT faced by academic institutions in LMICs outlined above pose major constraints for these institutions in generating and transferring knowledge to support the COVID-19 response. These constraints do not bode well for the extraordinary additional challenges for KT in the COVID-19 pandemic context, which include (1) speed and scale, as the disease has spread quickly to all corners of the world; (2) severity, as overall 20% of cases are severe or critical, with a crude clinical case fatality rate currently of over 3%, increasing in older age groups and in those with certain underlying conditions; and (3) societal and economic disruption, as shocks to health and social care systems and measures taken to control transmission have had broad and deep socioeconomic consequences [8]. Generating evidence around this last challenge, particularly to inform health systems and the economic and social response, may be in demand but also seen as less urgent than public health measures and clinical management, thus creating an uneven distribution of knowledge that could leave many inequities unaddressed or even exacerbated.

Additionally, the increasing role of social media has created the perfect breeding ground for myths, fake news, and conspiracy theories, some of which can be life-threatening [9, 10]. The COVID-19 pandemic has thus boosted the demand for knowledge to support decision-making at multiple levels. However, the pandemic has also unveiled the low level of readiness of academic institutions to conduct KT activities, particularly in the context of a complex health emergency.

## Policy windows, opportunities, and challenges

The pandemic opens policy windows in which policy actors tend to be more receptive of knowledge to inform their actions. For urgent complex decisions, many of which must be made within a few days, these often provide a series of windows which are open for brief periods to certain actors, sometimes with conflicting interests or views. This for instance may take form as a sudden invitation to take part in a ministerial videoconference, organized within a few hours after the invitation has been received, in preparation for a presidential cabinet meeting. For less urgent complex decisions, these could take form as opportunities to carry out a range of studies, which may encompass systematic reviews, rapid appraisal procedures, Delphi surveys, mathematical modelling, or even implementation research. This could emerge, for example, as a request from a government agency to document lessons learned for future pandemic preparedness policy. Notably, most academic institutions in LMICs are much less prepared to seize these policy window opportunities for urgent decisions, given the constraints highlighted above.

The resources and capacities required for effective KT are also often underestimated and underappreciated. During the pandemic, significant resources are being invested in research and development activities, particularly those aiming to develop new tools needed for COVID-19 response (such as rapid tests, diagnostic tests, drugs, prophylaxis, vaccines, ventilators). These financial resources in many settings are available to academic institutions, even in some LMICs. In contrast, there are limited, if any, budget allocations to support much needed KT activities. Most academic institutions in LMICs rely on low-cost media for KT, such as conducting virtual webinars, often haphazardly, and without strategic considerations. The accessibility of these webinar technologies has led to a surge in the number of webinars available, often exceeding the point of saturation.

Academic institutions in LMICs have also been unprepared to engage the public through social media. Many LMIC academic institutions and their scientists have limited presence in the social media or few resources allocated to communications. The few who actively engaged media often could not deliver their messages effectively or were misquoted, and some even experienced bullying when they dove into controversial/divisive subjects [11]. The lack of a solid body of evidence on COVID-19-related issues has aggravated this situation. COVID-19-related evidence quickly becomes out of date as the science progresses. Efforts to rapidly synthesize emerging evidence have also been problematic. Many COVID-19 reviews have flaws, failing to reliably separate useful signals from the scientific noise, and the reliability of these reviews has often been further compromised by questionable methodology. This may be because too few of the review authors, particularly from LMICs, have appropriate training and experience [7]. Moreover, there seems to be confusion about what constitutes a 'rapid review', which methods are appropriate, and what decision-makers should expect when requesting rapid reviews. Similar phenomena were documented many years ago when there was an explosion of evidence briefs for policy [12].

In the absence of sound scientific advice, non-experts are able to fill this vacuum with opinions which can be promoted more broadly than subtle academic voices. The muted voice of the academic community in LMICs can contribute significantly to irrational policy decisions for COVID-19 response. KT preparedness among LMICs’ academic institutions should be a critical part of efforts and financing to strengthen preparedness for future global health security threats.

## Conclusions

While global funding organizations have shifted priorities to support COVID-19 research and response, similar resources have not been allocated towards efforts that translate emerging findings to decision-makers. Academic institutions in LMICs, already heavily engaged in COVID-19 research, are poised to take advantage of unprecedented opportunities to engage with policy-makers but need structural, systemic, and strategic support to do so. We echo the call made by Stewart et al. [6] in their recent commentary in *The Lancet* that global health funders should consider this need and make long-term institutional investments in LMICs that address urgent needs, future preparedness and institutional capacity for KT.

## Data Availability

Not applicable.

## Abbreviations

KT: Knowledge translation
LMICs: Low- and middle-income countries
